# A randomized trial of tigecycline versus ampicillin-sulbactam or amoxicillin-clavulanate for the treatment of complicated skin and skin structure infections

**DOI:** 10.1186/1471-2334-12-297

**Published:** 2012-11-12

**Authors:** Peter Matthews, Marc Alpert, Galia Rahav, Denise Rill, Edward Zito, David Gardiner, Ron Pedersen, Timothy Babinchak, Paul C McGovern

**Affiliations:** 1 Department of Family Medicine, Department of Health, Mpumalanga, Middelburg, 1050, South Africa; 2 Central Montgomery Surgical Associates, 1057 South Broad Street, Lansdale, PA, 19446, USA; 3 Chaim Sheba Medical Center, Infectious Disease Unit, Tel Hashomer, Ramat Gan, 52621, Israel; 4 Pfizer Inc, Collegeville, PA, 19426, USA

**Keywords:** Tigecycline, Glycylcycline, cSSSI, Skin and skin structure infection

## Abstract

**Background:**

Complicated skin and skin structure infections (cSSSIs) frequently result in hospitalization with significant morbidity and mortality.

**Methods:**

In this phase 3b/4 parallel, randomized, open-label, comparative study, 531 subjects with cSSSI received tigecycline (100 mg initial dose, then 50 mg intravenously every 12 hrs) or ampicillin-sulbactam 1.5-3 g IV every 6 hrs or amoxicillin-clavulanate 1.2 g IV every 6-8 hrs. Vancomycin could be added at the discretion of the investigator to the comparator arm if methicillin-resistant *Staphylococcus aureus* (MRSA) was confirmed or suspected within 72 hrs of enrollment. The primary endpoint was clinical response in the clinically evaluable (CE) population at the test-of-cure (TOC) visit. Microbiologic response and safety were also assessed. The modified intent-to-treat (mITT) population comprised 531 subjects (tigecycline, n = 268; comparator, n = 263) and 405 were clinically evaluable (tigecycline, n = 209; comparator, n = 196).

**Results:**

In the CE population, 162/209 (77.5%) tigecycline-treated subjects and 152/196 (77.6%) comparator-treated subjects were clinically cured (difference 0.0; 95% confidence interval [CI]: -8.7, 8.6). The eradication rates at the subject level for the microbiologically evaluable (ME) population were 79.2% in the tigecycline treatment group and 76.8% in the comparator treatment group (difference 2.4; 95% CI: -9.6, 14.4) at the TOC assessment. Nausea, vomiting, and diarrhea rates were higher in the tigecycline group.

**Conclusions:**

Tigecycline was generally safe and effective in the treatment of cSSSIs.

**Trial registration:**

ClinicalTrials.gov NCT00368537

## Background

Skin and skin structure infections are classified as complicated (cSSSIs) if the infection has spread to the deeper tissues, surgical intervention is required, or the patient has a comorbid condition (e.g. diabetes mellitus) that complicates response to treatment [1]. Although most community-acquired skin infections are caused by *Staphylococcus aureus* and *Streptococcus pyogenes*, cSSSIs may have a diverse bacterial etiology depending on the clinical diagnosis, anatomic location, and healthcare setting [2-5]. The availability of therapies effective against a variety of pathogens including community-acquired methicillin-resistant *S. aureus* (CA-MRSA) may be desirable with certain cSSSIs.

Tigecycline is an intravenous expanded broad-spectrum glycylcycline with *in vitro* activity against pathogens associated with cSSSI [6,7]. The *in vitro* activity includes Gram-positive and Gram-negative organisms (except *Pseudomonas* spp.), anaerobes, atypical organisms, and some multidrug-resistant pathogens [8-11]. Tigecycline is not affected by classical tetracycline resistance mechanisms and has no cross-resistance with common resistance mechanisms in other classes of antibiotics; however, resistance to tigecycline via non-specific multidrug efflux pumps has been demonstrated [12].

Tigecycline has previously been found to be non-inferior to the combination of vancomycin and aztreonam in adults for the treatment of cSSSI in two randomized, double-blind, phase 3 trials [13,14]. Here, the efficacy and safety of tigecycline in hospitalized patients was compared with that of ampicillin-sulbactam or amoxicillin-clavulanate, which are commonly used in the treatment of cSSSI.

## Methods

A phase 3b/4, randomized, open-label, comparative study was conducted in subjects with cSSSI between September 2006 and September 2008 at 77 centers worldwide. The protocol (Pfizer Inc, data on file) was approved by the ethics committee of each participating center and written informed consent was obtained from each subject prior to enrollment. The study was block randomized by site (block size of 4); allocation sequence was not provided to investigators. Investigators were responsible for subject enrollment. Subjects were centrally randomized by computer at a 1:1 ratio to receive either tigecycline or comparator for a minimum of 4 days and a maximum of 14 days. Subjects assigned to tigecycline received 100 mg IV followed by 50 mg every 12 hrs. Subjects assigned to the comparator received ampicillin-sulbactam 1.5 to 3 g IV every 6 hrs or amoxicillin-clavulanate 1.2 g IV every 6 to 8 hrs. Vancomycin 1 g IV every 12 hrs could be added to the comparator regimen if infection with MRSA was suspected or confirmed within the first 72 hrs of enrollment. The aminopenicillin/β-lactamase inhibitor and vancomycin (by serum levels) could be dose-adjusted per local guidelines. If culture results failed to isolate MRSA, vancomycin could be discontinued.

### Subjects

Subjects were eligible for the study if they were 18 yrs or older and required hospitalization for cSSSI including deep soft tissue infection (e.g., cellulitis ≥10 cm, requiring surgery/drainage or with complicated underlying disease [e.g., diabetes mellitus, peripheral vascular disease, peripheral neuropathy, or venous insufficiency]), major abscess, infected ulcers, or burns <5% body surface area. The subject also had to have at least two of the following signs and symptoms: fever, erythema, drainage/discharge, swelling/induration, localized warmth, pain/tenderness, and white blood cell count >10,000/mm^3^ or >10% immature bands.

Subjects were excluded if they had an uncomplicated skin infection (e.g., simple abscesses, folliculitis, impetiginous lesions, furunculosis, or superficial cellulitis) or an infection that could be treated by drainage or wound care alone. Subjects with necrotizing infections, osteomyelitis, likely amputation, retained devices, and chronic infected (>1 week) ulcers were excluded. Subjects with chronic diabetic foot infection (DFI) were excluded. Subjects with hepatic disease or creatinine clearance <30 mL/min were also excluded from the study. Subjects could not receive more than 24 hrs of prior antibiotic therapy, unless they were a failure on prior antibiotic therapy (i.e., received prior antibiotics for ≥3 days with no improvement in the clinical signs and symptoms of infection). Subjects who were prior antibiotic failures required a baseline culture prior to the first dose of antibiotic in order to be eligible for the study. Subjects with hypersensitivity to study medications or subjects with concomitant infections that required treatment with another antimicrobial agent were excluded. Subjects with suspected or known *Pseudomonas aeruginosa* infections were excluded unless *P. aeruginosa* was part of a polymicrobial infection and the subject was continuing to improve.

Randomized subjects were included in the intent-to-treat (ITT) population. Subjects who received at least one dose of study drug were included in the modified ITT (mITT) or safety population. Subjects meeting the minimal disease criteria were included in the clinical modified ITT (c-mITT) population. The clinically evaluable (CE) population comprised c-mITT subjects who met all inclusion/exclusion criteria and had a clinical response of either cure or failure at the test-of-cure (TOC) assessment 8-50 days following the end of therapy (EOT). Subjects with confirmed baseline isolates were included in the microbiologic-modified ITT (m-mITT) and microbiologically evaluable (ME) population, respectively. ME subjects also had to have at least one isolate susceptible to both test articles.

### Efficacy and safety evaluations

A clinical response of cure, failure, or indeterminate was determined by each investigator at the EOT and TOC assessment. Subjects were cured if they had resolution or improvement of symptoms such that no further antibiotic therapy was required. Subjects were considered failures if they had an inadequate response requiring additional antibiotic therapy or additional surgical therapy to eradicate the infection. Subjects were considered failures if they switched to oral therapy or were considered indeterminate if clinical response could not be determined. Microbiologic efficacy (eradication/persistence) was determined at the subject and isolate levels. Adverse events (AEs) were collected from the time of informed consent throughout the study period.

When clinically appropriate, specimens from the site of infection were obtained at baseline and during the study. Blood cultures were obtained at baseline and thereafter when clinically indicated, and organism identification and susceptibility testing were confirmed at a central laboratory (Covance Clinical Laboratories, Indianapolis, IN). Minimum inhibitory concentrations (MIC) were determined by broth microdilution and by Kirby-Bauer disk diffusion (tigecycline only) according to procedures published by the Clinical and Laboratory Standards Institute (CLSI) [15,16]. CA-MRSA was defined in this study as the presence of staphylococcal cassette chromosome mec (SCC*mec*) type IV typing as determined by multiplex polymerase chain reaction.

### Statistical analysis

Statistical analysis was performed by the Clinical Biostatistics Department, Quintiles (Bloemfontein, South Africa). The primary efficacy endpoint was the clinical response in the CE population at the TOC. Assuming an evaluability rate of at least 60%, approximately 500 subjects were planned to be enrolled to obtain 300 CE subjects. Assuming the two treatments to be equally effective, with favorable clinical response rates (i.e., cure rates) of 80% at the TOC visit, 150 subjects per treatment group were required to ensure with 90% power that the lower bound of a two-sided 95% confidence interval (CI) for the true difference in efficacy (tigecycline minus comparator) corrected for continuity did not exceed -15%.

The noninferiority of tigecycline compared with comparator was evaluated for clinical and microbiological response by using a two-sided 95% CI for the true difference in efficacy (tigecycline minus comparator). Noninferiority was concluded if the lower limit of the two-sided CI was greater than -15%. Baseline variables, AEs, and health outcome data were compared with Fisher’s exact test, one-way analysis of variance with treatment as a factor, or log-rank test as appropriate. For subpopulation analyses, an adjusted difference between treatment groups was used. Comparisons involving small sample sizes were analyzed by the method of Wilson [17], corrected for continuity. The “exact” method of Clopper and Pearson [18] was used to determine the two-sided 95% CI for a single proportion. Throughout this article, significant refers to a p value <0.05.

## Results

**Figure 1:**
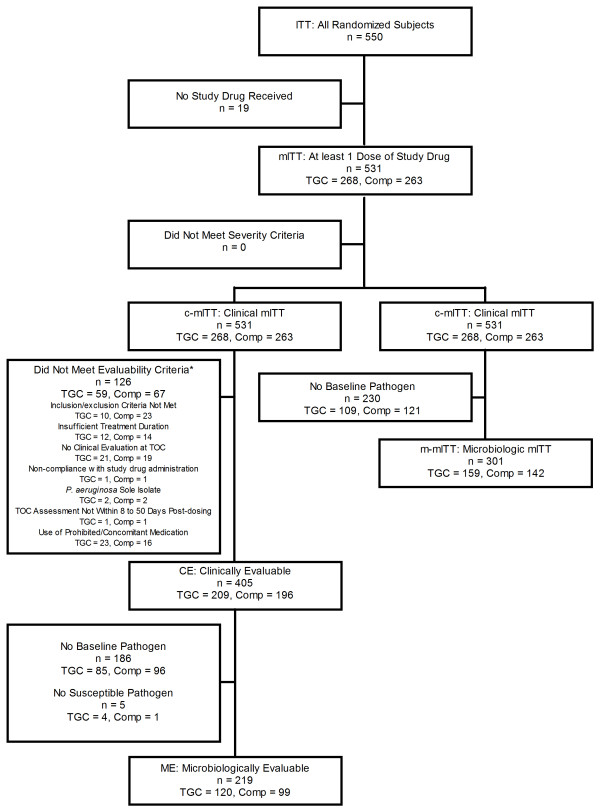
**Analysis population.** Footnote: *Subjects could be excluded for more than 1 reason. ITT, intent-to-treat; mITT, modified intent-to-treat; TGC, tigecycline; Comp, comparator. c-mITT, clinical modified intent-to-treat; TOC, test-of-cure; m-mITT, microbiologic-modified intent-to-treat; CE, clinically evaluable; ME, microbiologically evaluable.

A total of 550 subjects were enrolled and randomized. Nineteen subjects did not receive study drug and all subjects met severity of infection criteria (Figure 1). Fifty-nine tigecycline and 67 comparator subjects did not meet evaluability criteria (p = 0.360). No clinical evaluation at TOC, use of prohibited/concomitant medication, inclusion/exclusion criteria not met, and insufficient treatment duration were the most common reasons for non-evaluability. Five subjects were excluded from the ME population because of isolates not susceptible to both test articles; in each case resistance was present for the comparator drug.

### Patients’ characteristics

Baseline characteristics including demographics, comorbid conditions, and clinical diagnoses were similar for both treatment groups (Table 1). Thirty-two percent of patients had diabetes mellitus. Cellulitis was the most common diagnosis (63% of the total infections and 92% of subjects with deep soft tissue infection). The most common sites of infection were the lower extremity (61.6%) and upper extremity (16.2%). Lesion size was ≥10 cm in length or width (where anatomically feasible) for 74.9% of subjects. Spontaneous infection (60.3%), trauma (20.7%), and previous surgery (7.5%) were the most common causes of infection. Mean duration of treatment in both treatment groups was 8 days. The number of subjects who required any baseline procedure (e.g., debridement, incision, and drainage) for the infection was similar (tigecycline 41.8% versus comparator 46.4%; p = 0.295). Thirty-eight percent of comparator subjects received at least one dose of adjunctive vancomycin therapy.

**Table 1:** **Demographic and baseline characteristics of the mITT population**^**‡**^

	**Tigecycline (n = 268)**	**Comparator (n = 263)**
Age, years, mean (SD)	51.1 (16.11)	51.54 (16.90)
Males (%)	60.8	64.3
Ethnic origin (%)		
White	52.6	55.5
Asian	22.4	22.1
Black	16.4	15.2
Hispanic	5.6	4.9
Other	3.0	2.3
Weight, mean (SD)	87.8 (30.6)	90.4 (34.8)
Creatinine clearance, mL/min, mean (SD)	114.23 (60.73)	112.88 (59.94)
Comorbid conditions (%)		
Diabetes mellitus	31.7	32.7
Peripheral vascular disease	14.2	11.0
IV drug use	4.5	4.6
Prior antibiotic failure (%)	20.5	22.4
Clinical diagnosis, n (%)*		
Deep soft tissue infection^†^	186 (69.4)	176 (66.9)
Major abscesses	47 (17.5)	60 (22.8)
Infected ulcers	31 (11.6)	26 (9.9)

*Includes 3 burns and 1 “other” diagnosis in tigecycline subjects and 1 burn in comparator subjects. ^†^Includes cellulitis (168 and 166 patients in the tigecycline and comparator groups, respectively), wound infections (8 and 6 patients, respectively), bites (8 and 4 patients, respectively), and IV catheter infections (2 and 0 patients, respectively). ^‡^No statistically significant differences between groups. *mITT*, modified intent-to-treat; *SD*, standard deviation.

### Clinical outcomes

Tigecycline met the predefined statistical criteria for non-inferiority in the primary efficacy (CE) population. Tigecycline cured 162/209 (77.5%) subjects and the comparator cured 152/196 (77.6%) subjects (difference 0.0; 95% CI: -8.7, 8.6). Similar results were seen in the other study populations including the MITT population where 70.1% of tigecycline-treated subjects and 68.8% of comparator-treated subjects were cured (difference 1.3; 95% CI: -6.9, 9.5). Clinical responses in both treatment groups were similar based on clinical diagnoses and comorbidities (Table 2). Tigecycline efficacy in the CE population (77.5%) was similar to comparator subjects who did (72.7%) and did not (80.0%) receive vancomycin adjunctive therapy. No differences in cure rates were observed between treatment groups in either those who did or did not require baseline procedures (data not shown).

**Table 2:** Clinical success rates by study population at the test-of-cure visit

	**Tigecycline CE population N = 209 (%)**	**Comparator CE population N = 196 (%)**	**Difference (Tigecycline vs Comparator)**^**†**^**(95% CI)**
Clinical diagnoses*			
Deep soft tissue infections	114/150 (76.0)	103/132 (78.0)	-2.0 (-12.6, 8.5)
Major abscess	30/36 (83.3)	34/43 (79.1)	4.3 (-15.5, 24.0)
Infected ulcers	17/22 (77.3)	15/20 (75.0)	2.3 (-28.3, 32.9)
**Other populations**			
Diabetes mellitus	46/60 (76.7)	46/66 (69.7)	7.0 (-10.0, 24.0)
Peripheral vascular disease	20/27 (74.1)	16/20 (80.0)	-5.9 (-34.4, 22.5)
Prior antibiotic failure	27/35 (77.1)	26/34 (76.5)	0.7 (-22.1, 23.5)

*Includes 2 CE patients with burns: 1 tigecycline cure and 1 comparator failure; ^†^Calculated using asymptomatic method corrected for continuity. *CE*, clinically evaluable; *CI*, confidence interval.

Clinical cure rates for pathogens commonly found in cSSSI were comparable between the two groups (Table 3). Cure rates for subjects with methicillin-susceptible *S. aureus* (MSSA) were higher than cure rates for subjects with MRSA. In the ME population, clinical cure rates for concomitant bacteremia with a skin pathogen were 5/8 (62.5%) in tigecycline-treated subjects and 4/5 (80.0%) for comparator-treated subjects.

**Table 3:** Clinical cure rate at the isolate level: selected baseline isolates at test-of-cure visit (ME population)*

**Isolate**	**Tigecycline**		**Comparator**	
	**n/N**	**%**	**n/N**	**%**
*Staphylococcus aureus*	54/71	76.1	49/61	80.3
MRSA	25/36	69.4	21/29	72.4
CA-MRSA	17/25	68.0	17/23	73.9
MSSA	29/35	82.9	28/32	87.5
*Streptococcus* spp.	30/36	83.3	15/25	60.0
*S. pyogenes*	8/11	72.7	5/5	100
*S. agalactiae*	9/9	100	3/6	50.0
*S. anginosus* group^a^	8/9	88.9	3/8	37.5
*Enterobacter cloacae*	5/8	62.5	1/1	100
*Enterococcus* spp*.*^b^	5/7	71.4	6/6	100
*Escherichia coli*	10/13	76.9	7/8	87.5
*Klebsiella pneumoniae*	5/5	100	5/6	83.2
*Proteus* spp*.*^c^	4/7	57.1	2/2	100
*Pseudomonas aeruginosa*	5/7	71.4	2/2	100

*No statistically significant differences between groups. *ME*, microbiologically evaluable; *MRSA*, methicillin-resistant *Staphylococcus aureus*; *CA-MRSA*, community-acquired MRSA; *MSSA*, methicillin-susceptible *Staphylococcus aureus*. ^a^*Streptococcus anginosis, Streptococcus intermedius, Streptococcus constellatus;*^b^*Enterococcus faecalis, Enterococcus faecium;*^c^*Proteus mirabilis, Proteus penneri, Proteus vulgaris* group.

### Microbiology and microbiologic response

Within the m-mITT population, *S. aureus* was isolated in 58% of subjects with half (29%) demonstrating methicillin-resistance. Seventy-four percent of MRSA were CA-MRSA with SCC*mec* type IV. *S. aureus* MIC_90_ value for vancomycin was 1 μg/mL with 97% of isolates having an MIC of ≥1 μg/mL. Tigecycline MIC_90_ values for *S. aureus* and *Streptococcus pyogenes* were 0.25 μg/mL and 0.06 μg/mL, respectively. Gram-negatives (excluding *Pseudomonas *spp.), anaerobes, and polymicrobial infections were identified in 42%, 5%, and 43% of m-mITT subjects, respectively. Polymicrobial infections involving at least one Gram-positive and one Gram-negative pathogen occurred in 19% of infections. During therapy, reduced susceptibility to tigecycline was not identified in any isolate.

At the subject level, the eradication rates at the TOC assessment for the ME population were 79.2% in the tigecycline treatment group and 76.8% in the comparator treatment group (difference 2.4%; 95% CI: -9.6, 14.4). Within the ME population, the clinical response rate for tigecycline-treated subjects with monomicrobial infections was 86.4% compared with 72.4% for comparator-treated subjects (difference 13.9; 95% CI: -1.8, 29.7). For polymicrobial infections, the clinical response rate for tigecycline-treated subjects was 72.2% compared with 85.4% for comparator-treated subjects (difference -13.1; 95% CI: -31.4, 5.1).

### Health outcomes

Mean days of primary inpatient hospitalization did not differ between the treatment groups (tigecycline 8.54 versus comparator 8.78; p = 0.650). The proportion of subjects requiring readmission to the hospital was similar between the tigecycline and comparator treatment group (9.3% versus 8.0%; p = 0.645). Both groups were comparable in other post-hospital resource utilization such as nursing home and home health services (data not shown).

### Safety/tolerability

Seventy percent of subjects reported at least one treatment-emergent AE (TEAE). A total of 201/268 (75.0%) subjects who received tigecycline and 169/263 (64.3%) subjects who received comparator (p = 0.008) reported TEAEs. Excluding nausea, vomiting, and diarrhea, there was no difference in the rate of TEAEs between the two treatment groups (p = 0.326). Table 4 shows the TEAEs that occurred in ≥3% of subjects. Nausea and vomiting events were almost exclusively mild to moderate in severity (NCI grades 1 and 2). No investigator reported *Clostridium difficile*-associated diarrhea.

**Table 4:** Treatment-emergent adverse events in ≥3% of mITT subjects

**Adverse Event**	**Tigecycline**		**Comparator**	
	**N = 268**		**N = 263**	
	**n (%)**		**n (%)**	
Nausea*	117	(43.7)	44	(16.7)
Vomiting*	64	(23.9)	14	(5.3)
Diarrhea*	39	(14.6)	14	(5.3)
Constipation	17	(6.3)	22	(8.4)
Dyspepsia	17	(6.3)	7	(2.7)
Headache	20	(7.5)	22	(8.4)
Pain^†^	15	(5.6)	18	(6.8)
Abdominal pain	17	(6.3)	7	(2.7)
Fever	9	(3.4)	6	(2.3)
Chest pain	6	(2.2)	8	(3.0)
Insomnia	22	(8.2)	17	(6.5)
Anxiety	9	(3.4)	7	(2.7)
Dizziness	9	(3.4)	6	(2.3)
Hypokalemia*	6	(2.2)	17	(6.5)
Pruritis	15	(5.6)	16	(6.1)
Hypertension	8	(3.0)	9	(3.4)
Anemia	8	(3.0)	7	(2.7)

*Significant between-group difference (p < 0.05). ^†^Pain was a general category for any pain without a more specific category (e.g., abdominal pain). *mITT*, modified intent-to-treat.

Overall, 16/268 (6.0%) tigecycline subjects and 8/263 (3.0%) comparator subjects discontinued study drug because of an AE (p = 0.143) with no significant differences between treatment groups in the frequency of any single AE leading to discontinuation of study drug. Nine tigecycline subjects and three comparator subjects discontinued study drug because of nausea and/or vomiting. Serious AEs occurred in 14.2% and 11.0% of tigecycline and comparator subjects, respectively (p = 0.297). Eleven subjects died during the study: six subjects in the tigecycline treatment group and five subjects in the comparator treatment group. The majority of deaths were not attributed to the primary infection under study; furthermore, four tigecycline deaths and one comparator death occurred >30 days after the start of therapy. All of these deaths were assessed by the investigators as not related to study medication.

## Discussion

Skin and skin structure infections frequently result in hospitalization with significant morbidity and mortality. Failure to initiate appropriate empiric medical therapy can result in longer hospital stays, increased costs, and mortality [19-21]. Recent increases in ambulatory visits and hospitalization due to cSSSI have been attributed to the increase of CA-MRSA [22,23]. In this study, the most common pathogen was *S. aureus*, which was isolated in more than half of all subjects in the m-mITT population. Half of the *S. aureus* isolates demonstrated methicillin resistance, and 74% of them were CA-MRSA. The majority of all *S. aureus* isolates had vancomycin MICs of ≥1 μg/mL consistent with prior observations of vancomycin MIC creep [24,25]. However, a more diverse microbiological spectrum of pathogens in skin infections has previously been reported and was observed in this study [2,3]. Therefore, empiric treatment of hospitalized subjects with cSSSI may require broad spectrum antibiotic regimens and may require activity against MRSA.

A working group in South Africa has published guidelines on the appropriate use of tigecycline and has provided clinical scenarios that may be useful to clinicians [26]. In particular they have suggested that directed therapy against polymicrobial infections that include resistant pathogens such as extended spectrum β-lactamases and empiric therapy for subjects at risk of such infections would be supported. Patients with prior antibiotic exposure or failure, renal insufficiency, or β-lactam allergy may also benefit from tigecycline.

In this study, tigecycline was effective as empiric monotherapy in the treatment of hospitalized subjects with cSSSI. Tigecycline monotherapy was non-inferior to a commonly prescribed antibiotic for cSSSI, the aminopenicillin/β-lactamase inhibitor (±vancomycin). These results confirmed and are consistent with the results of two previous randomized, double-blind, active-controlled, multinational, multicenter phase 3 trials [13,14]. The combined clinical cure rate in the previous two studies was 86.5% in the tigecycline group versus 88.6% in the vancomycin/aztreonam group, (difference -2.1%; 95% CI: -6.8 to 2.7) [27].

Both tigecycline and comparator were generally well tolerated. Tigecycline has increased gastrointestinal AEs consistent with other tetracycline antibiotics; however, the severity of the nausea and vomiting were almost exclusively mild to moderate and few subjects required treatment discontinuation. An increase in all-cause mortality has been observed in the tigecycline clinical program; however, a difference was not observed in this study or in the cSSSI indication (risk difference 0.7; 95% CI: -0.5, 1.9) [28]. Overall, tigecycline was demonstrated to be safe in this clinical trial and the safety profile is consistent with prior cSSSI trials.

Two strengths of this study were that more than 60% of subjects presented with cellulitis and 20% were prior antibiotic failures. In a recent paper on the justification for noninferiority margins in cSSSI trials, the greatest benefit for antimicrobial therapy was seen in patients with cellulitis and erysipelas [29]. In a recent health outcomes paper, Edelsberg and colleagues demonstrated worse outcomes and increased resource utilization in cSSSI patients who were prior antibiotic failures [19]. In the current study, tigecycline compared favorably with the comparator regimen in subjects who had cellulitis and in subjects who were prior antibiotic failures (Table 2). The major limitation of this study is its open-label design and the potential bias on the outcomes. However, the outcomes are consistent with prior double-blinded studies in cSSSI.

This trial and the two previous clinical trials in cSSSI support the efficacy and safety of tigecycline use in this patient population [13,14]. Tigecycline has been included in the Surgical Infections Society’s updated guidelines for the management of cSSIs, specifically for the treatment of rapidly progressive soft tissue infections due to *S. aureus* and MRSA [20]. The Infectious Disease Society of America’s (IDSA) guidelines on the treatment of MRSA acknowledged the approved use of tigecycline within this indication but did not include tigecycline in its recommendations because of available MRSA-active alternatives and the increase in all-cause mortality noted in the tigecycline clinical program [30]. Recently, the IDSA guidelines on DFIs [31] has listed tigecycline as a suggested empiric regimen despite tigecycline not meeting primary study endpoints in its DFI clinical trial.

## Conclusions

In conclusion, tigecycline was non-inferior to ampicillin-sulbactam or amoxicillin-clavulanate with or without vancomycin in the treatment of cSSSI. This trial and the two previous clinical trials in cSSSI support the efficacy and safety of tigecycline use in this patient population. Given its broad spectrum *in vitro* activity, tissue penetration, and clinical trial results, tigecycline continues to offer an effective and safe alternative option, where appropriate, in the empiric treatment of hospitalized patients with cSSSI.

## Competing interests

This study was sponsored by Wyeth Research, which was acquired by Pfizer Inc in October 2009. These results were presented in part at the 20th European Congress of Clinical Microbiology and Infectious Diseases, Vienna, Austria, 2010. D. Rill, P. McGovern, and E. Zito are employees of Pfizer Inc, USA. T. Babinchak and D. Gardiner are former employees of Pfizer Inc and Wyeth Research.

## Authors’ contributions

PM, MA, and GR were study investigators and helped draft the manuscript. DR, EZ, DG, RP, TB, and PG participated in study design and coordination and helped draft the manuscript. All authors read and approved the final manuscript.

## Authors’ information

Study Investigators, Sites, and Number of Subjects Recruited

Marc Alpert, Central Montgomery Surgical Association, Lansdale, PA 19446, USA (28); Peter Armstrong, Dwight D Eisenhower Army Medical Center, Fort Gordon, GA 30905-5650, USA (1); Charles Bailey, Aliso Viejo, CA 92656, USA; German Berbel, HealthFirst Medical Group Research, Fort Worth, TX 76104, USA (14); Jack Bernstein, Dayton Veteran Affairs Medical Center, Dayton, OH 45428, USA; Jose Bordon, Providence Hospital Clinical Research Center, NE Washington, DC 20017, USA (8); Lou Ann Bruno-Murtha, Cambridge Health Alliance, Somerville, MA 02143, USA (5); Russell Caprioli, Long Island Jewish Medical Center, New Hyde Park, NY 11040, USA (4); Kathleen Casey, Jersey Shore University Medical Center, Neptune, NJ 07754, USA (3); Tom Chiang, VA NJ Healthcare System, East Orange, NJ 07018, USA (5); Allan Churukian, eStudySite, San Diego, CA 92114, USA; William Flynn, ECMC Department of Surgery, Buffalo, NY 14215, USA (1); Donald Graham, Springfield Clinic LLP, Springfield, IL 62701, USA (11); Zijun Hao, St. Elizabeth Regional Medical Center, Lincoln, NE 68510, USA (17); Kenneth Kalassian, Emory University Hospital, Atlanta, GA 30322, USA; Richard Kohler, Wishard Hospital, Indianapolis, IN 46202, USA; Juliet Lee, The Medical Faculty Associates, Washington, DC 20037, USA; William Leeds, Topeka, KS 66606, USA (2); Christopher Lucasti, South Jersey Infectious Disease, Somers Point, NJ 08244, USA (22); Gregory Malanoski, AMS Infectious Disease, Elmira, NY 14905, USA; Tien Ko, LBJ General Hospital Surgery Department, Houston, TX 77026, USA (8); Venkat Minnaganti, Infectious Disease Specialists of Central Illinois, Decatur, IL 62526, USA (2); Miguel Mogyoros, Kaiser Permanente, Denver, CO 80205, USA (2); Bill Morgan, Harris Methodist Fort Worth Hospital, Fort Worth, TX 76104, USA; Charles Moss, Moss and Geuder Surgical Group, Hackensack, NJ 07601, USA; Satish Muluk, Allegheny General Hospital, Pittsburgh, PA 15212, USA (5); Rekha Murthy, Los Angeles, CA 90048, USA (1); William O’Riordan, eStudysite, San Diego, CA 92114, USA (8); Francis Pien, Honolulu, HI 96814, USA (13); Hiram Polk, University of Louisville, Louisville, KY 40292, USA (1); James B. Augustinsky, Naperville, IL 60563, USA (10); Michelle Salvaggio, Infectious Diseases Institute Clinical Trials Unit, Oklahoma City, OK 73104-5068, USA; Leon Smith, Saint Michael’s Medical Center, Newark, NJ 07102, USA; Raymond Smith, Stratton VA Medical Center, Albany, NY 12208, USA; R. Scott Stienecker, Regional Infectious Diseases-Infusion Center, Lima, OH 45801, USA (20); Byungse Suh, Section of Infectious Diseases, Philadelphia, PA 19140, USA (4); Jose Vazquez, Henry Ford Health System, Detroit, MI 48202, USA (24); Dennis E. Weiland, Scottsdale, AZ 85259, USA (5); Mireya Wessolossky, UMass Medical School/UMass Memorial Center, Worcester, MA 01655, USA (5); Jonathan Zenilman, Johns Hopkins Bayview Medical Center, Baltimore, MD 21224, USA (1); Carl Abraham, Jonesboro, AR 72401, USA (6); Richard Nathan, Idaho Falls, ID 83404, USA (5); Phillip Sanchez, Southeastern Clinical Research Consultants, Orlando, FL 32804, USA; Ian Baird, Remington-Davis, Inc., Columbus, OH 43215, USA (4); Charles Callahan, Clinical Research Center of Indian River Medical Center, Vero Beach, FL 32960, USA; Christian G. Schrock, Infectious Diseases Minneapolis Ltd., Minneapolis, MN 55422, USA (6); William Lau, Honolulu, HI 96813, USA (1); Markian R. Bochan, Infectious Disease of Indiana, Indianapolis, IN 46280, USA; Michael Somero, Research Office, Palm Springs, CA 92262, USA (1); Stanley R. Klein, Harbor-UCLA Medical Center, Torrance, CA 90509, USA (4); Charles Bellows III, Tulane University Health Science Center, New Orleans, LA 70112, USA; Annick D’Hooghe, St-Elisabeth Ziekenhuis, Antwerpen, 2000, Belgium; Françoise Ceulemans, Center Hospitalier Regional de Namur, Belgium; Jacques Gaillat, Center Hospitalier General d’Annecy, Annecy Cedex, 74011, France; Bernard Garo, CHU Hopital de la Cavale Blanche, 29609 Brest Cedex, France; Christian Eckmann, Universitaetsklinikum, 23538 Lubeck, Germany (9); Joerg Haier, Molecular Biology Laboratory, 48149 Munster, Germany (3); Fredy Suter, U.O. Malattie Infettive, Bergamo, 24128, Italy; Aldo Bertani, A.O.U. Ospedali Riuniti Umberto I G.M Lancisi-G. Salesi, 60126 Torrette di Ancona, Italy (5); Francisco Acin, Hospital Universitario de Getafe Servicio de Cirugia Vascular Planta 4B, 28905 Getafe (Madrid), Spain (4); Manuel E. Jiménez-Mejías, Hospital Universitario Virgen del Rocio, Sevilla, 41013, Spain; Ignacio Blanes, Hospital Universitario Dr. Peset, Valencia, 46017, Spain; Dolores Sousa Regueiro, Comolejo Hospitalaro, 15006 A Coruna, Spain (2); Nedim Cakir, Dokuz Eylul University Medical Faculty Clinical Microbiology and Infectious Diseases, Izmir 35340, Turkey; Rabin Saba, Akdeniz University Medical Faculty, 07070 Antalya, Turkey; Michael Giladi, Infectious Disease Unit, Tel-Aviv, 64239, Israel (5); Galia Rahav, Chaim Sheba Medical Center, Ramat Gan, 52662, Israel (18); Souha Kanj-Sharara, American University of Beirut, Beirut, 110 32090, Lebanon (5); Abdulhakeem Okab Ahmed al Thaqafi, King Abdulaziz Medical City-Western Region Infectious Disease Department, Jeddah, 21423, Saudi Arabia (5); Wai-Man Ng, Queen Mary Hospital, Hong Kong (1); Andrew Burd, Prince of Wales Hospital, Shatin, New Territories, Hong Kong (2); Utkrant Kurlekar, Deenanath Mangeshkar Hospital and Research Center, Maharashtra, India (8); N. Raghupathi Rao, Apollo Hospitals, Andhra Pradesh, India (4); T. Devarajan, Apollo First Med Hospitals, Tamil Nadu State, India; Junyong Choi, Yonsei University Medical Center Severance Hospital, Medical School, Seoul, 120-752, South Korea (4); Yeonsook Kim, Chungnam National University Hospital, Daejeon, 301-721, South Korea (7); Hyunjoo Pai, Hanyang University Hospital, Seoul, 133-791, South Korea (13); Yoon-Soo Park, Gachon Medical School Gil Medical Center, Incheon, 405-760, South Korea (14); Suresh Kumar, Hospital Sungai Buloh, 47000, Selangor, Malaysia (1); Ting Soo Chow, Hospital Pulau Pinang, 10990 Penang, Malaysia (5); Armando Crisostomo, Philippine General Hospital, Manila, 1000, Philippines (6); Alex Erasmo, University of Santo Tomas Hospital, Manila, 1008, Philippines (7); Alex Erasmo, Jose R. Reyes Memorial Medical Center, Manila, 1014, Philippines (8); Jenny Low, Singapore General Hospital, Singapore, 169608, Singapore (8); M.M. Basson, Tiervlei Trial Center, Bellville, 7531, Cape Town, South Africa (7); Johannes Breedt, Eugene Marais Medical Village, Pretoria, South Africa (8); P.A. Matthews, Middelburg Hospital, Middelburg, 1050, South Africa (21); D.P. Ross, St. Mary’s Hospital, Durban, South Africa (5); His-Hsun Lin, E-Da Hospital, Kaohsiung County, Taiwan (7); Chun-Hsing Liao, Far Eastern Memorial Hospital, Taipei County, Taiwan (9); Hsiang-Chi Kung, National Taiwan University Hospital, Yunlin County, 640, Taiwan (3); Vitoon Chinswangwatanakul, Siriraj Hospital, Bangkok, 10700, Thailand (6); Kumthorn Malathum, Ramathibodi Hospital, Rajathevee, Bangkok 10400, Thailand; Terapong Tantawichien, Faculty of Medicine, Pathumwan, Bangkok 10330, Thailand; Sergio Ricardo Filho Penteado, Hospital Universitario Evangelico de Curitiba, CEP: 80730-150, Brazil; Fernando Cardoso, Hospital Universitario Clementino Fraga Filho, Ilha do Fundao, Rio de Janeiro, Brazil (1); Roosevelt Fajardo Gomez, Fundacion Santa Fe do Bogota, Bogota DC, Colombia; David Fernandez Velazquez, Instituto Nacional de Ciencias Medicas y Nutricion, Delegacion Tlalpan CP 14000, Mexico; Juan Carlos Tinoco-Favila, Durango, Mexico (4); Andre Poirier, Centre Hospitalier Régional de Trois-Rivières, Québec, G8Z 3R9, Canada (17); Louis Valiquette, CRC – C.H.U.S. - Hôpital Fleurimont, Sherbrooke Québec, J1H 5N4, Canada (2); Karl Weiss, Hôpital Maisonneuve-Rosemont, Montréal, Québec, H1T 2 M4, Canada (7); Doria Grimard, Center de Santé et des Services Sociaux de Chicoutimi, Chicoutimi Québec, G7H 5H6, Canada (19); John M.A. Embil, Winnipeg Health Sciences Center, Winnipeg Manitoba, R3A 1R9, Canada (3); Steven E. Sanche, Royal University Hospital, Saskatoon Saskatchewan, S7N 0W8, Canada (1); Ken Smith, Discovery Clinical Services, Victoria British Columbia, V8T 5G4, Canada (1); Sylvain Chouinard, Institut Universitaire de Cardiologie et de Pneumologie de Québec, Québec, G1V 4G5, Canada (9); Patrick Dolcé, Center de Santé et de Services Sociaux de Rimouski-Neigette, Rimouski Québec, G5L 5T1, Canada.

## Pre-publication history

The pre-publication history for this paper can be accessed here:

http://www.biomedcentral.com/1471-2334/12/297/prepub
